# Esophageal squamous cell carcinoma in a patient with spinal cord injury complicated by achalasia: a case report

**DOI:** 10.3389/fonc.2026.1764934

**Published:** 2026-04-13

**Authors:** YuHang Zhang, XiaoMing Li

**Affiliations:** 1 Department of Thoracic Surgery, North Sichuan Medical College, Nanchong, China; 2 Department of Thoracic Surgery, Guangyuan Central Hospital, Guangyuan, China

**Keywords:** achalasia, case report, esophageal squamous cell carcinoma (ESCC), rare, spinal cord injury (SCI)

## Abstract

The progression from spinal cord injury(SCI) to secondary achalasia and subsequent esophageal squamous cell carcinoma (ESCC) is exceptionally rare. We report the case of a 51-year-old female who developed progressive dysphagia seven years after a T6-level spinal injury. Barium swallow and endoscopic examinations revealed the classic “bird’s beak sign” and a suspicious esophageal lesion. Given the patient’s paraplegic condition and associated anesthetic risks, endoscopic intervention was contraindicated. Following evaluation by a multidisciplinary team, a combined thoracoscopic-laparoscopic esophagectomy with cervical anastomosis, lymph node dissection, and pyloroplasty was performed. Postoperative pathology confirmed ESCC (pT2N0M0, Stage IIB). This case suggests a potential pathological sequence of “SCI → achalasia → ESCC”: SCI may induce or exacerbate achalasia through neurogenic mechanisms, while prolonged esophageal stasis likely elevates the risk of malignant transformation. Additionally, symptom masking due to paraplegia and procedural challenges often contribute to diagnostic delays, adversely affecting prognosis. This report highlights the need for individualized surveillance protocols and multidisciplinary intervention pathways in SCI patients with long-standing achalasia to improve clinical outcomes.

## 1 Introduction

Spinal Cord Injury (SCI) is defined as structural or functional impairment of the spinal cord resulting from trauma, disease, or degeneration, leading to motor, sensory, and autonomic dysfunction below the level of injury (1). Achalasia is an idiopathic primary esophageal motility disorder characterized by impaired relaxation of the lower esophageal sphincter (LES) and loss of esophageal peristalsis, primarily due to the reduction or absence of myenteric neurons (2–4). Previous studies indicate a substantially increased prevalence of esophageal dysmotility in SCI patients due to chronic neural impairment (5). This chronic dysmotility can predispose individuals to achalasia, which in turn is a known risk factor for esophageal squamous cell carcinoma (ESCC) due to prolonged esophageal stasis (6). A 2023 multicenter retrospective study reported an annual achalasia incidence of approximately 1.10 per 100,000 person-years (7), while large database analyses estimate ESCC incidence at 15.4 per 100,000 person-years (8). However, literature review reveals notably scarce reports of SCI-induced achalasia with coexisting ESCC. Here, this report outlines the individualized treatment for an adult woman with T6 SCI, with concurrent achalasia and ESCC.

## 2 Case report

A 51-year-old female was admitted to the hospital due to intermittent postprandial vomiting for 7 years, dysphagia for 8 months, and worsening symptoms over the past month. Her medical history was significant for a traumatic T6-level spinal cord injury 15 years prior, resulting in complete paraplegia. On admission, her nutritional status was poor, with a BMI of 17.5 kg/m². Physical examination revealed complete loss of sensation and motor function below the T6 level, with muscle strength in both lower limbs graded as 0 and absence of deep tendon and superficial reflexes, including the abdominal wall and anal reflexes. There was mild tenderness over the T5-T6 spinous processes, while other vital signs remained stable. Laboratory tests showed no significant abnormalities.

**Figure 1:**
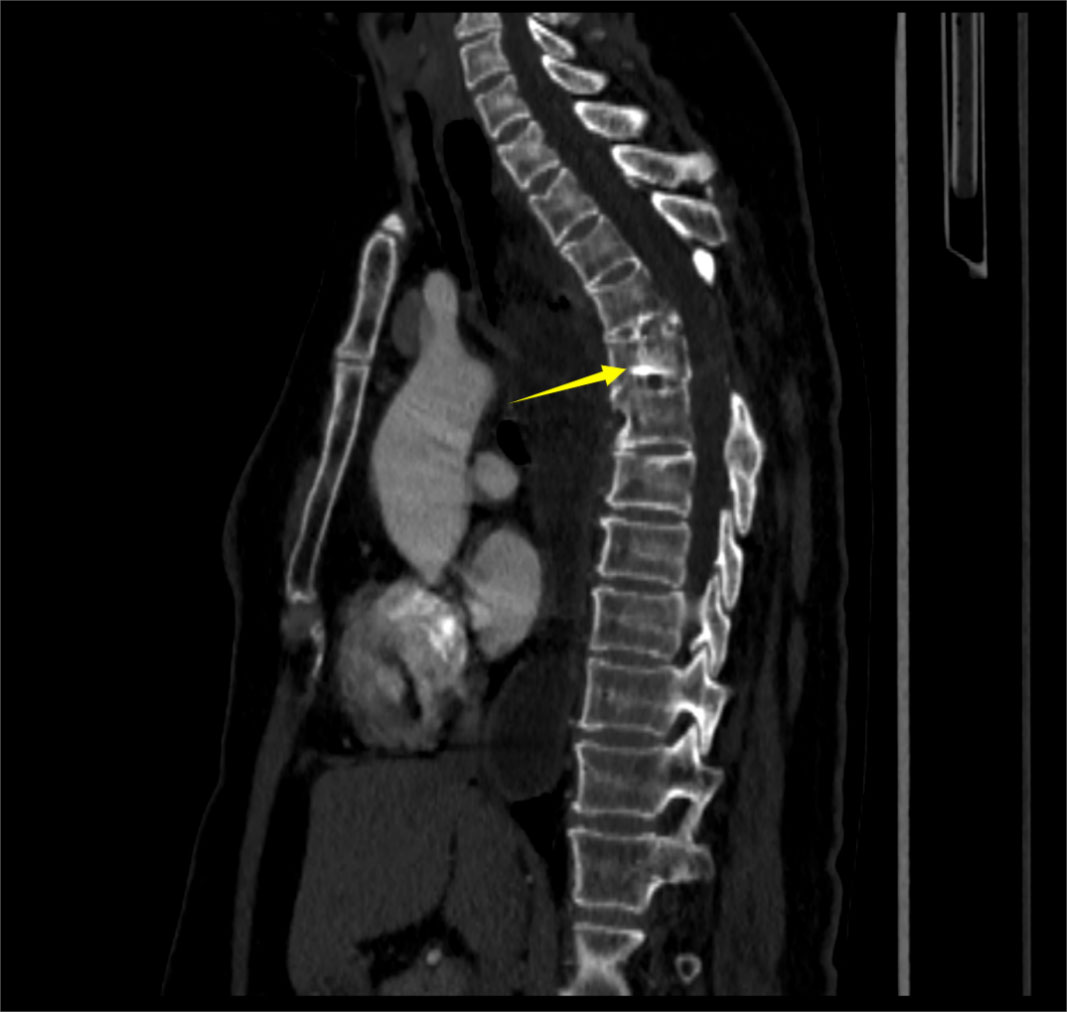
CT scan of the thoracic spine. Sagittal image at the T5–6 level shows heterogeneous density within the spinal canal (arrowhead), with disorganized bone structure and partial destruction of the vertebral bodies and posterior elements (arrow), consistent with chronic post-traumatic changes.

**Figure 2:**
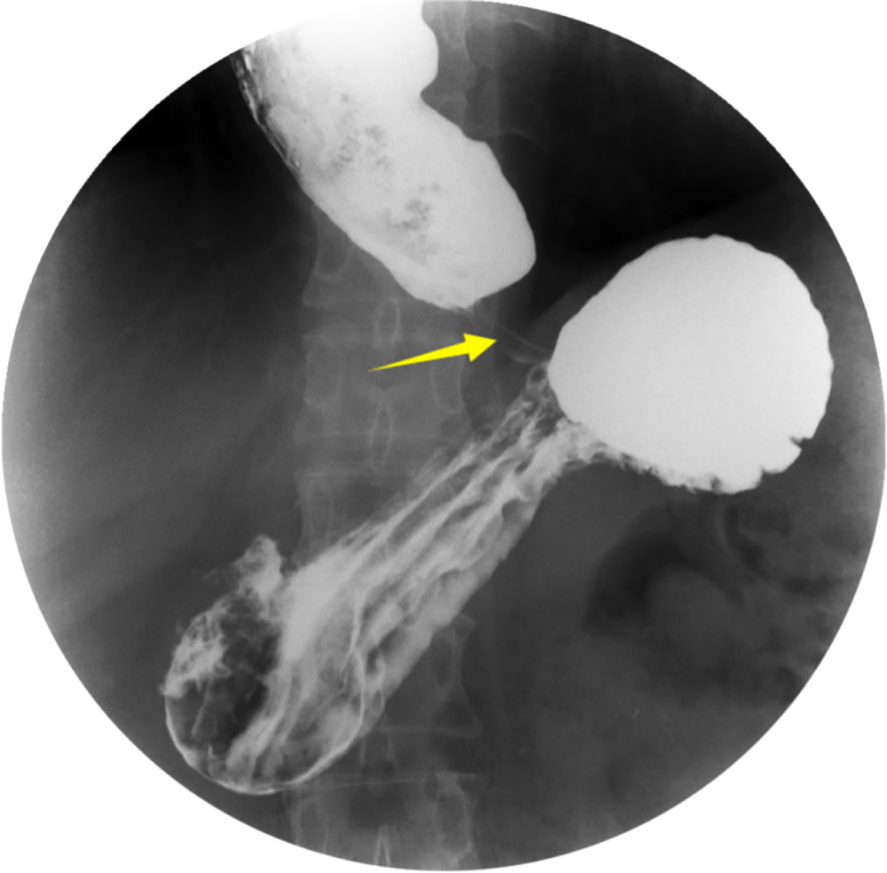
Upper gastrointestinal barium swallow. The image demonstrates a dilated esophagus with retained barium and a smooth, tapered, concentric narrowing at the level of the esophagogastric junction, producing the classic “bird’s beak” sign (arrow), characteristic of achalasia.

**Figure 3:**
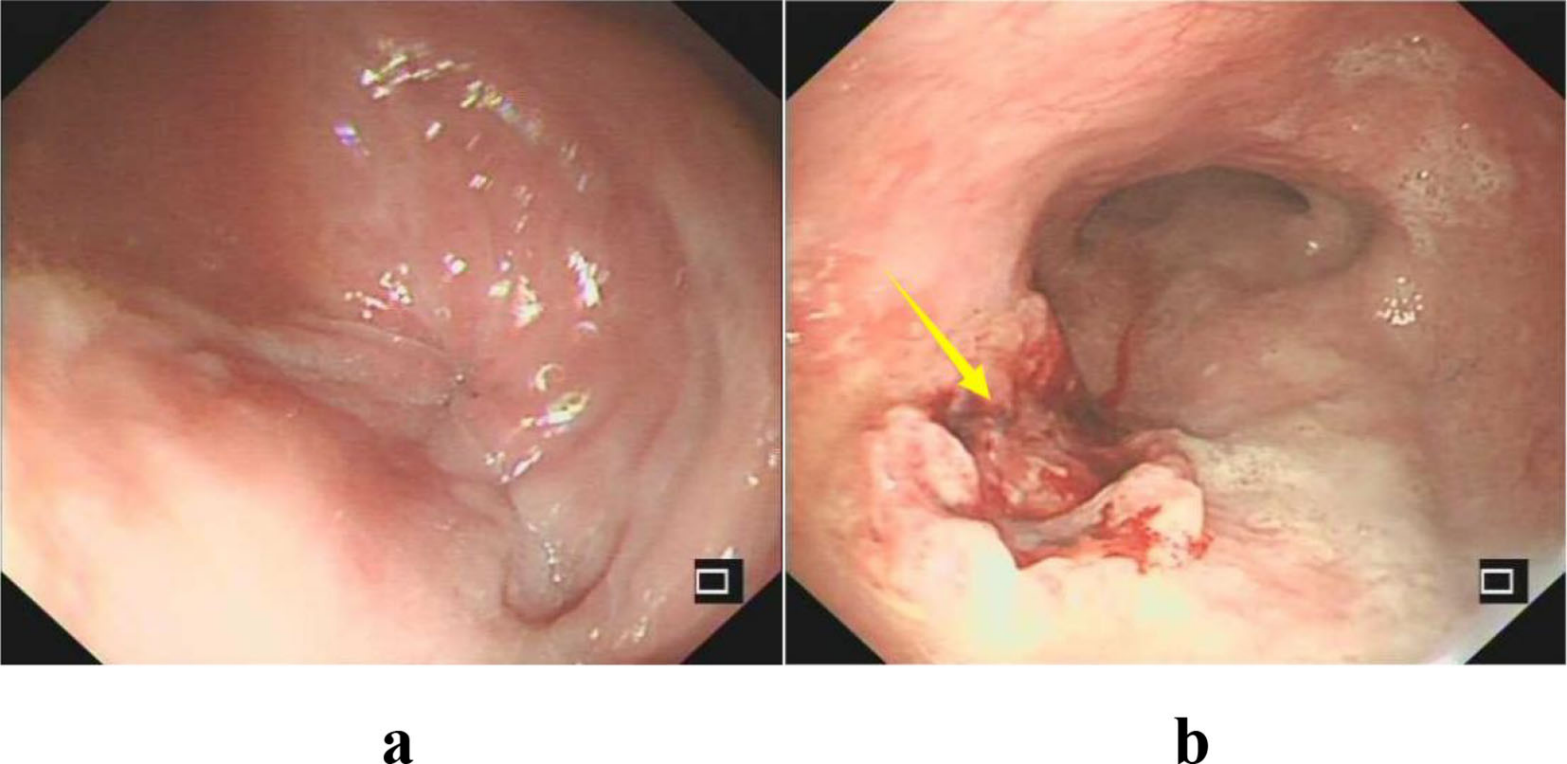
Esophagogastroduodenoscopy (EGD) findings. **(a)** Endoscopic view of the esophagogastric junction showing a puckered, rosette-like appearance of the mucosa, typical of achalasia. **(b)** A view of the mid-esophagus, approximately 21 cm from the incisors, reveals an ill-defined, friable, and irregular mass (arrows) measuring approximately 3 cm × 3 cm, highly suspicious for carcinoma.

To evaluate the structural changes at the site of her old spinal injury and rule out any compressive pathology contributing to her new symptoms, a CT scan was performed. It indicated heterogeneous density within the spinal canal at the T5–6 level, with disorganized bony structures and partial destruction of the vertebral bodies and attachments (**Figure 1**). Given her progressive dysphagia, an upper gastrointestinal barium meal examination was chosen as a non-invasive initial test to assess esophageal morphology and motility. This revealed the classic “bird’s beak” sign, strongly suggestive of achalasia (**Figure 2**). Subsequently, an esophagogastroduodenoscopy (EGD) was performed to directly visualize the esophageal mucosa and obtain biopsies. EGD demonstrated a characteristic “esophageal rosette” appearance (**Figure 3a**), confirming the diagnosis of achalasia, and also identified an ill-defined, friable neoplasm approximately 3 cm × 3 cm in size, located about 21 cm from the incisors, which was highly suspicious for malignancy (**Figure 3b**).

To further characterize the esophageal motility disorder, high-resolution manometry (HRM) was considered but could not be performed due to the patient’s inability to maintain the required semi-recumbent position for an extended period and the high risk of aspiration. Consequently, a Chicago Classification subtype for achalasia could not be determined. Endoscopic ultrasound (EUS) was also planned for local tumor staging (T-stage) and to assess for any subepithelial invasion or adjacent lymph nodes. However, the procedure was aborted due to the significant tortuosity of the dilated esophagus and the presence of retained food debris, which made safe passage of the echoendoscope impossible. Preoperative CT imaging of the chest and abdomen was reviewed in detail by the multidisciplinary team. The scan revealed a mid-esophageal wall thickening corresponding to the known tumor, without clear evidence of invasion into adjacent mediastinal structures (cT3); no enlarged regional lymph nodes (cN0) or distant metastases (cM0) were identified.

Given the patient’s paraplegia, difficulty in maintaining positions for prolonged endoscopic procedures, and a high potential risk of respiratory insufficiency under sedation, a systemic evaluation indicated high risks for anesthesia and endoscopic interventions like endoscopic submucosal dissection (ESD) or peroral endoscopic myotomy (POEM). To address her severe dysphagia and poor nutritional status, which was a major concern, a duodenal feeding tube was placed under gastroscopy to ensure adequate nutritional intake. This was accompanied by symptomatic treatments such as acid suppression and gastric mucosal protection. The patient was encouraged to actively exercise lung function in preparation for further surgery. Two weeks later, after obtaining informed consent, the patient underwent “combined thoracoscopic and laparoscopic esophagectomy with left cervical esophagogastric anastomosis, mediastinal and abdominal lymph node dissection, and pyloroplasty”.

**Figure 4:**
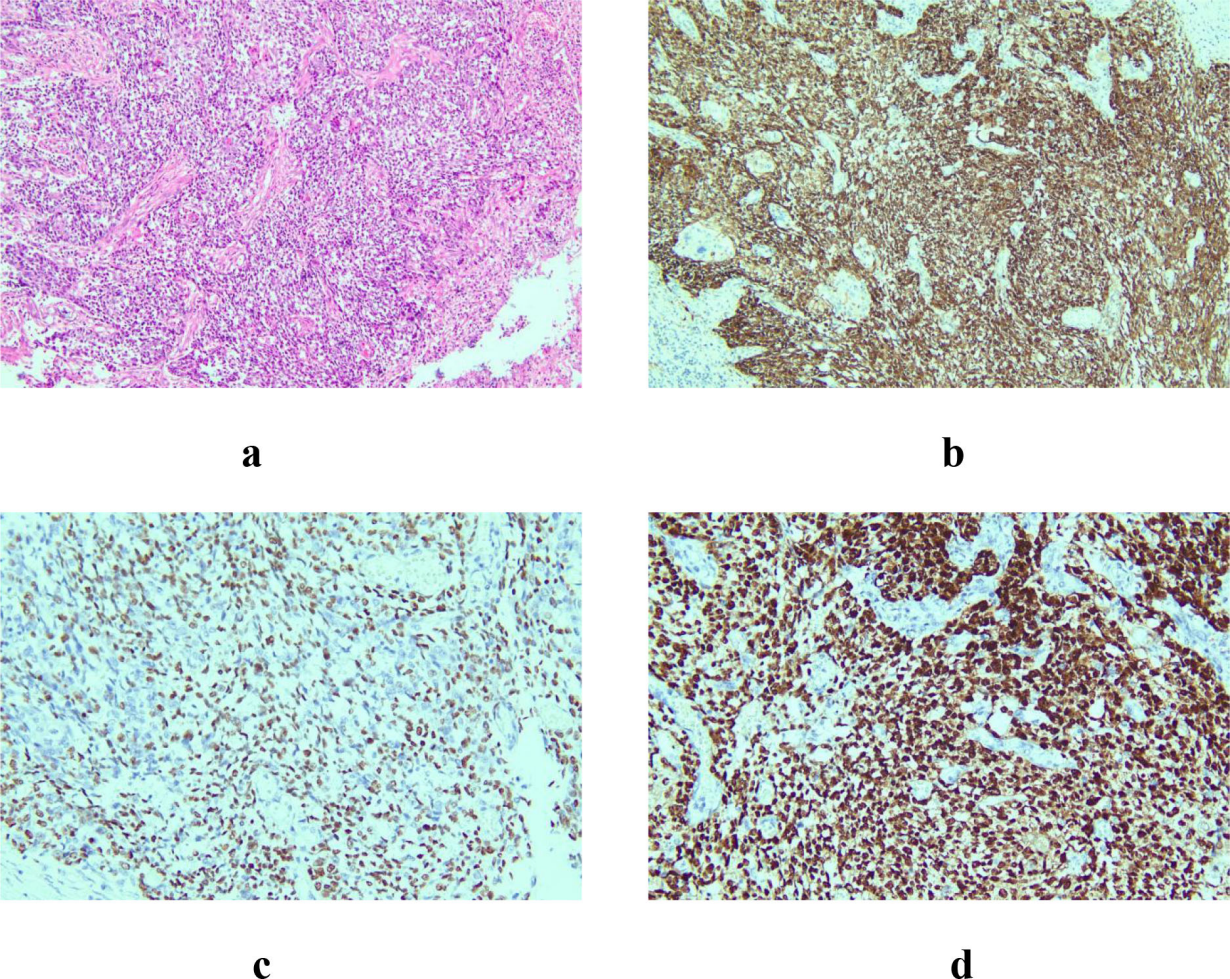
Postoperative histopathological and immunohistochemical analysis. **(a)** Hematoxylin and eosin (HE) staining shows infiltrating nests of squamous cell carcinoma confirming ESCC (original magnification x100). **(b)** Immunohistochemistry (IHC) shows strong and diffuse positivity for CKpan (AE1/AE3) in tumor cells (original magnification x100). **(c)** IHC shows strong nuclear positivity for P40 (original magnification x100). **(d)** IHC shows strong nuclear positivity for P53 (original magnification x100).

The surgery was performed electively once her nutritional status had slightly improved with tube feeding. Postoperatively, the patient’s bowel function returned on day 4, and she began oral intake on day 7. She was discharged on day 14 with complete resolution of dysphagia. The surgical margins were negative. Postoperative pathological examination confirmed esophageal squamous cell carcinoma. Histopathological and immunohistochemical analysis of the surgical specimen (**Figure 4**) showed: CKpan (AE1/AE3) (+), P40 (+), P53 (+, missense mutation), and Ki67 (+, approximately 80%). Clinical diagnosis: esophageal squamous cell carcinoma (pT2N0M0, stage IIB). Postoperatively, the patient was treated with two cycles of adjuvant chemotherapy using a regimen of albumin-bound paclitaxel (200 mg on days 1 and 8 of a 21-day cycle) plus cisplatin (50 mg on days 2 and 3). She tolerated the chemotherapy well without any reported adverse events or need for dose modification. Subjectively, the patient reported complete resolution of her pre-operative dysphagia and was satisfied with the improvement in her ability to eat. She was discharged and scheduled for follow-up at 1, 3, and 6 months, including clinical evaluation and imaging. However, she was lost to follow-up after the second cycle of chemotherapy, and no further outcome data are available. The key clinical events, including symptom progression, diagnostic investigations, and therapeutic interventions, are summarized in a timeline (**Table 1**).

**Table 1:** Timeline of key clinical events.

Date/timeframe	Clinical event
15 years prior to admission	Traumatic T6 spinal cord injury, resulting in complete paraplegia.
7 years prior to admission	Onset of intermittent postprandial vomiting. Initially attributed to neurogenic bowel dysfunction.
8 months prior to admission	Onset of progressive dysphagia to solids.
1 month prior to admission	Worsening of dysphagia and vomiting.
Admission (Day 0)	Hospital admission. Physical exam, labs, and imaging (CT, barium swallow) initiated. Identified as high-risk for endoscopy. BMI noted at 17.5.
Day 3	Esophagogastroduodenoscopy (EGD) with biopsy, confirming achalasia and a suspicious esophageal mass. Duodenal feeding tube placed.
Day 17	Elective combined thoracoscopic-laparoscopic esophagectomy with cervical anastomosis, lymph node dissection, and pyloroplasty.
Day 28	Postoperative pathology confirms ESCC (pT2N0M0, Stage IIB). Discharged. Scheduled for adjuvant chemotherapy.
Months 1-2 (Post-op)	Received two cycles of adjuvant chemotherapy (albumin-bound paclitaxel + cisplatin). Tolerated well. Patient lost to follow-up thereafter.

## 3 Discussion

In this case, the patient developed esophageal squamous cell carcinoma (ESCC) on the basis of long-standing spinal cord injury and achalasia, which is consistent with existing literature indicating that achalasia significantly increases the risk of esophageal cancer (9). The shared pathological basis lies primarily in prolonged esophageal stasis. This stagnant environment may promote bacterial overgrowth and the accumulation of nitrosamine carcinogens, which collectively act on the squamous epithelial cells of the esophageal mucosa, inducing dysplasia and carcinogenesis (9).Chronic inflammation from stasis-related esophagitis may further potentiate this malignant transformation.

### 3.1 Diagnostic challenges and differential diagnoses

The diagnosis in this patient was complicated by her underlying SCI. The initial symptom of vomiting was attributed for years to neurogenic bowel dysfunction, a common issue in paraplegic patients, leading to a significant delay in diagnosis. The differential diagnosis for her dysphagia included: (1) Achalasia: The “bird’s beak” sign on barium swallow and the “esophageal rosette” on endoscopy were highly characteristic, confirming this diagnosis. (2) Pseudoachalasia secondary to a tumor at the gastroesophageal junction: This was a critical consideration, especially after the endoscopic finding of a mass. However, the tumor was located in the mid-esophagus, not at the EGJ, and the classic “bird’s beak” sign made pseudoachalasia from a distal obstruction less likely. The final pathology confirmed a primary ESCC separate from the EGJ. (3) Benign peptic stricture: The patient had no significant history of reflux symptoms, and the smooth, tapering narrowing on barium swallow was more consistent with achalasia than an irregular peptic stricture. (4) Esophageal web or ring: These were ruled out by the barium study and endoscopy. The diagnostic process was further hindered by the patient’s sensory deficits, which may have blunted the early perception of dysphagia, causing her to present only when symptoms became severe.

### 3.2 Pathophysiological interplay between SCI, achalasia, and ESCC

SCI likely contributed to the pathogenesis of achalasia in this case through multifaceted mechanisms. The potential link between SCI and the development of achalasia warrants further exploration of the underlying neurobiology. Normal LES relaxation is primarily mediated by inhibitory neurons within the myenteric plexus, which release vasoactive intestinal peptide (VIP) and nitric oxide (NO). The pathophysiological hallmark of idiopathic achalasia is the selective loss of these inhibitory neurons, leading to unopposed excitatory (cholinergic) input and subsequent failure of LES relaxation (2). SCI, particularly at the thoracic level (T6-T10), disrupts not only somatic motor pathways but also critical autonomic circuits, including the parasympathetic supply to the distal gut via the vagus nerve and sacral parasympathetic nucleus. While a direct causal relationship remains speculative, it is plausible that chronic autonomic dysregulation following SCI could create a permissive or aggravating environment for the degeneration of the inhibitory myenteric neurons. This neurogenic insult might lower the threshold for developing achalasia in predisposed individuals or accelerate its progression (10, 11). The long-term stasis resulting from this neurogenic achalasia then establishes the carcinogenic environment that likely led to ESCC in our patient (6). Thus, we propose a potential three-stage pathological process of “SCI → neurogenic achalasia → ESCC”.

### 3.3 Treatment considerations in a complex patient

The management of this patient required a highly individualized, multidisciplinary approach. The treatment strategy was formulated through a formal multidisciplinary team (MDT) conference involving thoracic surgeons, gastroenterologists, medical oncologists, anesthesiologists, and radiologists. Given the dual pathology of advanced achalasia and biopsy-proven ESCC, the MDT considered several options: (1) neoadjuvant chemotherapy or chemoradiotherapy followed by restaging and surgery; (2) upfront surgery followed by adjuvant therapy if indicated; (3) definitive chemoradiotherapy alone. Neoadjuvant therapy was deemed less favorable due to the patient’s poor nutritional status (BMI 17.5) and the risk of further decompensation during treatment, which could potentially render her inoperable. Definitive chemoradiotherapy was considered a non-curative option for her resectable tumor. Therefore, the consensus was to proceed with upfront surgical resection as the primary curative treatment, with the goal of R0 resection. The choice of a combined thoracoscopic-laparoscopic approach was made to minimize surgical trauma and aid in postoperative recovery, given her baseline respiratory compromise from SCI. The surgical plan was discussed in detail, including the extent of lymph node dissection and the decision to perform a pyloroplasty to facilitate gastric emptying.

Standard endoscopic therapies for achalasia, such as POEM, were contraindicated due to her inability to tolerate prolonged procedures in specific positions and the high anesthetic risk. While POEM is highly effective for relieving obstruction (12), it carries a notable risk of post-procedural GERD (13, 14). In a patient with SCI, who may already have impaired gastric emptying and blunted reflux symptoms, severe GERD could be particularly dangerous and might theoretically increase cancer risk in the remaining esophagus. Therefore, despite its invasiveness, a surgical approach (esophagectomy) was chosen as it definitively treated both the cancer and the underlying motility disorder in a single procedure, eliminating the future risk of GERD-related complications in the native esophagus. This decision underscores the principle that for SCI patients with achalasia and ESCC, the risk-benefit analysis of interventions must heavily weigh the patient’s unique physiology and long-term safety.

### 3.4 Strengths and limitations

The primary strength of this report is the rare and compelling clinical triad of SCI, achalasia, and ESCC, which offers a unique opportunity to explore their potential pathophysiological connections. It highlights the critical need for heightened clinical awareness and individualized care in this specific patient population.

However, this study has several limitations. As a single-case report, the observed temporal association is insufficient to establish a definitive causal relationship between SCI, achalasia, and ESCC. The findings may not be generalizable to other patient populations. The retrospective nature of the data collection introduces potential for bias. We acknowledge several important diagnostic limitations in this case. The inability to perform HRM for achalasia subtyping and EUS for precise locoregional staging represents a significant gap in the diagnostic workup, primarily due to the patient’s physical limitations and anatomical challenges posed by the severely dilated esophagus. The lack of these investigations limits the granularity of our pathophysiological and oncological understanding of this specific case. Furthermore, the patient was lost to follow-up after two cycles of chemotherapy, and the absence of long-term follow-up imaging and clinical data prevents us from reporting on surgical durability, recurrence, or quality-of-life outcomes. Based on her pathological staging (pT2N0M0, Stage IIB), the prognosis following complete surgical resection with negative margins is generally favorable, with 5-year survival rates reported in the literature ranging from 40-60% for this stage. Standard follow-up would include regular clinical assessments, imaging, and endoscopic surveillance, but this could not be carried out in this case.

## Data Availability

The original contributions presented in the study are included in the article/Supplementary Material. Further inquiries can be directed to the corresponding author.

## References

[1] American Association of Neurological Surgeons . Clinical guidelines for management of acute spinal cord injury. Neurosurgery. (2021) 88:E1–E34.

[2] Pressman A Behar J . Etiology and pathogenesis of idiopathic achalasia. J Clin Gastroenterol. (2017) 51:195–202. doi: 10.1097/MCG.0000000000000780, PMID: 28009686

[3] Yadlapati R Kahrilas PJ Fox MR . Esophageal motility disorders on high-resolution manometry: Chicago classification version 4.0^©^. Neurogastroenterol Motil. (2021) 33:e14058. doi: 10.1111/nmo.14058, PMID: 33373111 PMC8034247

[4] Kuribayashi S Iwakiri K Shinozaki T . Clinical impact of different cut-off values in high-resolution manometry systems on diagnosing esophageal motility disorders. J Gastroenterol. (2019) 54:1078–82. doi: 10.1007/s00535-019-01608-3, PMID: 31388756

[5] Radulovic M SChilero GJ Yen C . Greatly increased prevalence of esophageal dysmotility observed in persons with spinal cord injury. Dis Esophagus. (2015) 28:699–704. doi: 10.1111/dote.12272, PMID: 25224683

[6] Tustumi F Bernardo WM da Rocha JRM . Esophageal achalasia: a risk factor for carcinoma. A systematic review and meta-analysis. Dis Esophagus. (2017) 30:1–8. doi: 10.1093/dote/dox072, PMID: 28859394

[7] Chinese Journal of Digestion Editorial Board . Multi-center retrospective study of achalasia in China. Chin J Dig. (2023) 43:657–64. doi: 10.3760/cma.j.cn311367-20230704-00299, PMID: 41912385

[8] Zheng R Zhang S Zeng H . Cancer incidence and mortality in China, 2016. J Natl Cancer Center. (2022) 2:1–9. doi: 10.1016/j.jncc.2022.02.002, PMID: 39035212 PMC11256658

[9] Low EE Demb J Shah SC . Risk of esophageal cancer in achalasia: A matched cohort study using the nationwide veterans affairs achalasia cohort. Am J Gastroenterol. (2024) 119. doi: 10.14309/ajg.0000000000002591, PMID: 37975607 PMC10994742

[10] Radulovic M SChilero GJ Yen C . Greatly increased prevalence of esophageal dysmotility observed in persons with spinal cord injury. Dis Esophagus. (2015) 28:699–704. doi: 10.1111/dote.12272, PMID: 25224683

[11] Holmes GM Blanke EN . Gastrointestinal dysfunction after spinal cord injury. Exp Neurol. (2019) 320:113009. doi: 10.1016/j.expneurol.2019.113009, PMID: 31299180 PMC6716787

[12] Ma O Brar K McCluskey S Morris-Janzen D Peabody J Turner S . Long-term outcomes after per-oral endoscopic myotomy versus laparoscopic Heller myotomy in the treatment of achalasia: a systematic review and meta-analysis. Surg Endosc. (2025) 39:5985–94. doi: 10.1007/s00464-025-11895-y, PMID: 40624423

[13] Ravi K Ramchandani M . POEM and GERD: prevalence, mechanisms, potential strategies for prevention, and management. Clin Gastroenterol Hepatol. (2022) 20:2444–7. doi: 10.1016/j.cgh.2022.05.040, PMID: 35839867

[14] Katsumi A Mori H Matsuura N . Concurrent diagnosis of superficial esophageal cancer and esophageal achalasia: A case report and literature review. DEN Open. (2025) 6:e70164. doi: 10.1002/deo2.70164, PMID: 40529107 PMC12171625

